# Triple Difficulties in Japanese Women with Hearing Loss: Marriage, Smoking, and Mental Health Issues

**DOI:** 10.1371/journal.pone.0116648

**Published:** 2015-02-04

**Authors:** Yoko Kobayashi, Nanako Tamiya, Yoko Moriyama, Akihiro Nishi

**Affiliations:** 1 Department of Health Services Research, Faculty of Medicine, University of Tsukuba, Ibaraki, Japan; 2 Research and Support Center on Higher Education for the Hearing and Visually Impaired, Tsukuba University of Technology, Ibaraki, Japan; 3 Yale Institute for Network Science, Yale University, New Haven, Connecticut, United States of America; 4 Department of Sociology, Yale University, New Haven, Connecticut, United States of America; Hamamatsu University School of Medicine, JAPAN

## Abstract

**Objective:**

To examine the consequences of early-onset hearing loss on several social and health measures and any related gender differences in Japanese populations.

**Methods:**

Data from a 2007 nationally representative cross-sectional household survey of 136,849 men and women aged 20 to 39 years were obtained (prevalence of self-reported hearing loss: 0.74%). We focused particularly on four social and health measures: employment status (employed/unemployed), marital status (married/unmarried), smoking behavior (yes/no), and psychological distress (K6 instrument: ≥ 5 or not). We examined the association of hearing loss for each measure using generalized estimating equations to account for correlated individuals within households.

**Findings:**

There was no significant association with employment status (*p* = 0.447). Men with hearing loss were more likely to be married, whereas women with hearing loss were less likely to be married (*p* < 0.001 for interaction). Although hearing loss was not associated with a current smoking status in men, women with hearing loss were more likely to be current smokers (*p* < 0.001 for interaction). Moreover, hearing loss was associated with psychological distress in men and women (both *p* < 0.001).

**Conclusion:**

These findings suggest that hearing loss is related to social and health issues in daily life, including a lower likelihood of marriage, more frequent smoking, and poorer mental health, especially in women. These issues may reflect a gap between the actual needs of women with hearing loss and the formal support received as a result of existing public health policies in Japan.

## Introduction

Hearing loss (or hearing impairment) is one of the most frequent types of disability [1]. Currently, more than 360 million people (5.3% of the worldwide population are estimated to have some degree of hearing loss [2]. Evidence suggests that hearing loss leads to a poorer quality of life, greater difficulty in social activities, and a higher level of social isolation [3]. Evidence also suggests that hearing loss is associated with lower household income, poor occupation status and lower educational attainment [4,5]. It is also known that there is an association with several health conditions, including smoking [6,7] and psychological distress [8–10]. The health and wellbeing of people with disabilities, including hearing loss, remains one of the top priorities in public and health policies [11].

The United Nations adopted the Convention on the Rights of Persons with Disabilities (CRPD) in 2006, which came into effect in 2008 [12]. The convention affirms that persons with disabilities have “the right to work on an equal basis with others” (Article 27 of CRPD), “the right to marry on the basis of free and full consent of the intending spouses” (Article 23), and “the right to the enjoyment of the highest attainable standard of health without discrimination” (Article 25). It also places emphasis on the importance of statistics and data collection in formulating and implementing necessary policies (Article 31). Thus, policy makers need to consider formulating and implementing evidence-based public and health policies.

Japan was the 140th country worldwide to ratify the CRPD in January 2014 (27th among the 34 OECD countries) [13–15]. Although Japan may have made substantial progress in changing its social norms and policies against people with disabilities because of the disability rights movements in the 1990s and 2000s [16], it still took Japan seven years to amend its domestic laws, including the Services and Supports for Persons with Disabilities Act, to follow the convention [13,17].

This delayed ratification may have had negative impacts on the living conditions of people with disabilities, which need to be examined carefully. However, evidence on the living and health conditions of people with hearing loss in Japan is scarce. A nationwide survey of people with disabilities, conducted in 2006, reported that 0.27% of the Japanese population (*n* = 350,000) had some degree of hearing loss [18]; however, this number was restricted to those having a physical disability certificate (average hearing loss > 70 decibels, equivalent to severe/profound hearing loss). Thus, many more people with mild to moderate hearing loss were not included in the target population of the survey or covered by the social welfare program under the Law for the Welfare of People with Physical Disabilities [19]. Indeed, about 6,000,000 people (4.70%) are estimated to receive hearing aid, however can’t receive a physical disability certificate (average hearing loss: under 70 decibels) in Japan [20] and the health needs and living arrangements of those who do not have the physical disability certificate have rarely been focused on or investigated [21–23].

Additionally, women with disabilities need special attention, as recognized the Convention: “women and girls with disabilities are subject to multiple discrimination” (Article 6) [12]. Several qualitative studies have reported that Japanese women with disabilities might be disadvantaged (e.g., lower income, poorer health, and shorter educational attainment) compared with men with disabilities [24]. However, there has been no reported quantitative study examining gender differences in the associations between hearing loss and social and health conditions.

Thus, the purposes of this study were (i) to examine the association of early-onset hearing loss with several social and health measures and (ii) to examine any gender differences in these associations, using a larger sample of residents in Japan. We aimed particularly to investigate the conditions of early-onset hearing loss among young working-age adults. Here, within this age range, people are usually faced with various important choices for their lives [25]. For example, they conduct a job search, enter the world of work, find a marriage partner, and discuss family planning with the partner. All the decisions here are usually the lifetime ones and require intensive communications, and thus usually these issues can be the source of daily hassles, stresses, and health-related behaviors [26]. Therefore, we examined the influence of early-onset hearing loss on these broad ranges of living conditions: employment status (socioeconomic situation), marital status (living arrangement), current smoking (health-related behavior), and psychological distress (mental health).

## Methods

### Study population

We used the Comprehensive Survey of the Living Conditions of People on Health and Welfare (LCPHW: *Kokumin Seikatsu Kiso Chosa*, http://www.mhlw.go.jp/english/database/db-hss/cslc.html), which was conducted by Japan’s Ministry of Health, Labour and Welfare (MHLW) in June 2007. The LCPHW is a population-based cross-sectional health survey known to be the best nationally representative dataset for various socioeconomic and health conditions. Among the 624,178 respondents who answered the questionnaire, we restricted the study population to those who answered a series of questions on subjective symptoms (38,566 individuals were excluded, and study participants reporting no subjective symptoms were included). We also restricted the study population to those aged 20 to 39 years of age. In the 2007 LCPHW, the question on hearing loss, one of the subjective symptoms, did not distinguish early-onset hearing loss from age-related hearing loss (presbycusis). A previous study showed that the prevalence of overall “hearing loss” increased markedly at 40 to 50 years of age, due to the increased number of cases of age-related hearing loss [27]. Because the initial manifestations of age-related hearing loss are not likely to be seen before 40 years of age [28] study participants younger than 40 years are expected to be at lowest risk of age-related hearing loss. Indeed, the 2007 LCPHW showed that the prevalence of those reporting hearing loss remained at the same level among those aged 20 to 39 years, and then increased after age 40 (0.8% for age 20 to 29, 0.7% for age 30 to 39, 1.1% for age 40 to 49, 5.0% for age 50 to 59, 11.1% for age 60 to 69, and 24.7% for age 70 years and older). Thus, we used 136,849 study participants whose ages ranged from 20 to 39 years in the 2007 LCPHW for further analyses. We obtained permission for secondary use of the 2007 LCPHW data. This study was approved by the Ethical Committee of the University of Tsukuba (#862).

### Measures

We used four outcome measures (employment status, marital status, smoking, and psychological distress), which did not substantially overlap with the state of hearing loss itself and which covered various aspects of social and health conditions in young working-age adults.

**Socio-demographic factors**. Regarding socio-demographic factors, age, gender, employment status, and marital status were determined. Employment status, which reliability and validity were examined [29] was measured by asking the study participants if they are currently working or not. Marital status, representing living arrangements, was measured by asking if they are currently married, never married, widowed, or separated, which reliability and validity was discussed and has been widely used [30–32]. We created a dichotomous indicator variable for marital status: married and unmarried (never married, widowed, or separated).

**Smoking behavior**. Smoking behavior was measured by asking the study participants if they do not smoke, smoke every day, smoke sometimes, or smoke but not within the past one month. The reliability and validity of self-reported smoking behavior were examined and grossly assured in multiple studies (e.g. [33,34]). We then dichotomized these into currently smoking (the second and third categories) and not currently smoking (the first and fourth categories) [35].

**Mental health status**. As a psychological distress measure, we used the Kessler-6 scale (K6). K6 has been used widely around the world [36]. A Japanese version of K6 has also been validated [37,38] and been widely used [32,39]. K6 is based on answers to six-item psychological distress questions, and we calculated the sum of the reported scores, ranging from 0 to 24. We created a dichotomous indicator variable representing psychological distress, based on a previously proposed threshold of K6 scores ≥ 5 (for potential mood/anxiety disorder in a Japanese sample) [37].

**Hearing loss**. Hearing status was assessed on the basis of subjective symptoms. The study participants were asked whether they had each of 41 items—subjective symptoms (e.g. fever, headache, constipation, anorexia, shoulder stiffness, incontinence, fracture). One of the items is hearing loss (they answered yes or no for “*kikoenikui*”; the literal translation of this Japanese term is “difficulty in hearing”). Self-reported hearing loss is a widely used measure in epidemiological research, and validation has been reported in multiple studies [40,41]. Previous studies indicated that self-reported hearing loss was grossly equivalent to the averaged pure-tone thresholds > 25 decibel hearing level (i.e., mild hearing loss) [42]. Although standard audiometric testing is an objective technique used to examine hearing ability, a subjective measure of hearing loss is also important for understanding how study participants feel their hearing difficulty affects their daily life activities [43].

**Statistical analysis**. First, the associations between hearing loss status and other variables were assessed separately using χ^2^ tests and Student’s t-tests. First, χ^2^ tests were used to determine whether hearing loss was associated with the dichotomous variables (i.e., gender; employment status: employed/unemployed; marital status: married/unmarried; smoking behavior: yes/no; and psychological distress: K6 5 or not). Additionally, Student’s t-tests were used for comparing the continuous variables (e.g., age). Furthermore, we examined the associations of hearing loss with these variables separately in men and women. Second, generalized estimating equations (logit link function with an unstructured working correlation matrix) were used to calculate odds ratios and standard errors, which accounted for potentially correlated observations within the same households [32]. In the models, the associations of hearing loss with each of the four outcome variables were examined by controlling for age and gender. Furthermore, we examined these associations separately in men and women. We also calculated *p*-values for the interaction term (gender hearing loss) to formally test the gender difference in the degrees of associations of hearing loss with the outcome variables. Odds ratios (ORs) with 95% confidence intervals (CIs) are presented. All analyses were performed using the SPSS software (ver. 21).

## Results

### Sample characteristics

Table 1 summarizes the basic characteristics of the study population aged 20 to 39 years (*n* = 136,849; mean age: 30.36 years, standard deviation: 5.6), among whom 78.7% were currently employed and 47.7% were married. Most of those who were unmarried were never married (48.2%), while being widowed (0.2%) or separated (3.3%) was relatively rare. Among the study participants, 35.0% were current smokers, and 30.9% reported mild or severe psychological distress (K6 5). In total, 1,012 individuals (0.74%) reported a symptom of hearing loss. Given that a recent national survey reported that 0.27% of the Japanese population had “certified” hearing loss [18], these results indicated that we had identified a larger proportion of individuals with mild or moderate hearing loss. The results of statistical tests on bivariate associations are also shown in Table 1.

**Table 1:** Basic characteristics of young working-age adults in the 2007 LCPHW (n = 136,849).

	Total		All						Men						Women					
			Hearing loss		No hearing loss		t-statistic		Hearing loss		No hearing loss		t-statistic		Hearing loss		No hearing loss		t-statistic	
			*n* = 1,012 (0.74%)		*n* = 135,837 (99.26%)				*n* = 415 (0.62%)		*n* = 66,762 (99.38%)				*n* = 597 (0.86%)		*n* = 69,075 (99.14%)			
	mean ± SD		mean ± SD		mean ± SD				mean ± SD		mean ± SD				mean ± SD		mean ± SD			
Age	30.36 ± 5.6		30.35 ± 5.7		30.36 ± 5.6		-0.04^†^		30.39±5.755		30.33±5.658		0.19^†^		30.39±5.734		30.39±5.625		-0.25^†^	
	N	%	n	%	n	%	Chi-square		n	%	n	%	Chi-square		n	%	n	%	Chi-square	
Gender							26.64	***												
Male	67,177	49.1	415	41.0	66,762	49.1														
Female	69,672	50.9	597	59.9	69,075	50.9														
																				
Occupation Status^#^							4.11	*					2.30						0.00	
No	29,025	21.3	241	24.0	28,784	21.3			55	13.3	7,269	11.0			186	31.4	21,515	31.3		
Yes	106,175	78.7	765	76.0	106,175	78.7			358	86.7	58,944	89.0			407	68.6	47,231	68.7		
																				
Marital Status							1.01						3.63						11.39	***
Married	65,305	47.7	467	46.1	64,838	47.7			202	48.7	29,387	44.0			265	44.4	35,451	51.3		
Never married	66,692	48.7	480	47.4	66,212	48.7			199	48.0	35,860	53.7			281	47.1	30,352	43.9		
Widowed	309	0.2	3	0.3	306	0.2			1	0.2	117	0.2			2	0.3	189	0.3		
Separated	4,543	3.3	62	6.1	4,481	3.3			13	3.1	1,398	2.1			49	8.2	3,083	4.5		
																			
Smoking^#^							5.35	*					0.42						47.86	***
No	85,849	65.0	608	61.5	85,241	65.1			204	50.0	31,003	48.4			404	69.7	54,238	81.0		
Yes	46,158	35.0	380	38.5	45,778	34.9			204	50.0	33,052	51.6			176	30.3	12,726	19.0		
																				
Psychological Distress^#^							690.59	***					328.74	***					352.61	***
No	87,544	69.1	279	29.6	87,265	69.4			118	30.3	44,007	71.9			161	29.2	43,258	67.0		
Yes	39,165	30.9	662	70.4	38,503	30.6			271	69.7	17,197	28.1			391	70.8	21,306	33.0		

Unmarked χ^2^ tests were used to examine the differences between those with and without hearing loss.

† Student’s t-tests were used to examine the differences between those with and without hearing loss.

# Study participants with missing values were excluded.

^¶^ The three categories were combined into the single category of “unmarried” for further analyses.

*** *p* < 0.001, ** *p* < 0.01, * *p* < 0.05.

### Multivariate analyses

**Fig 1:**
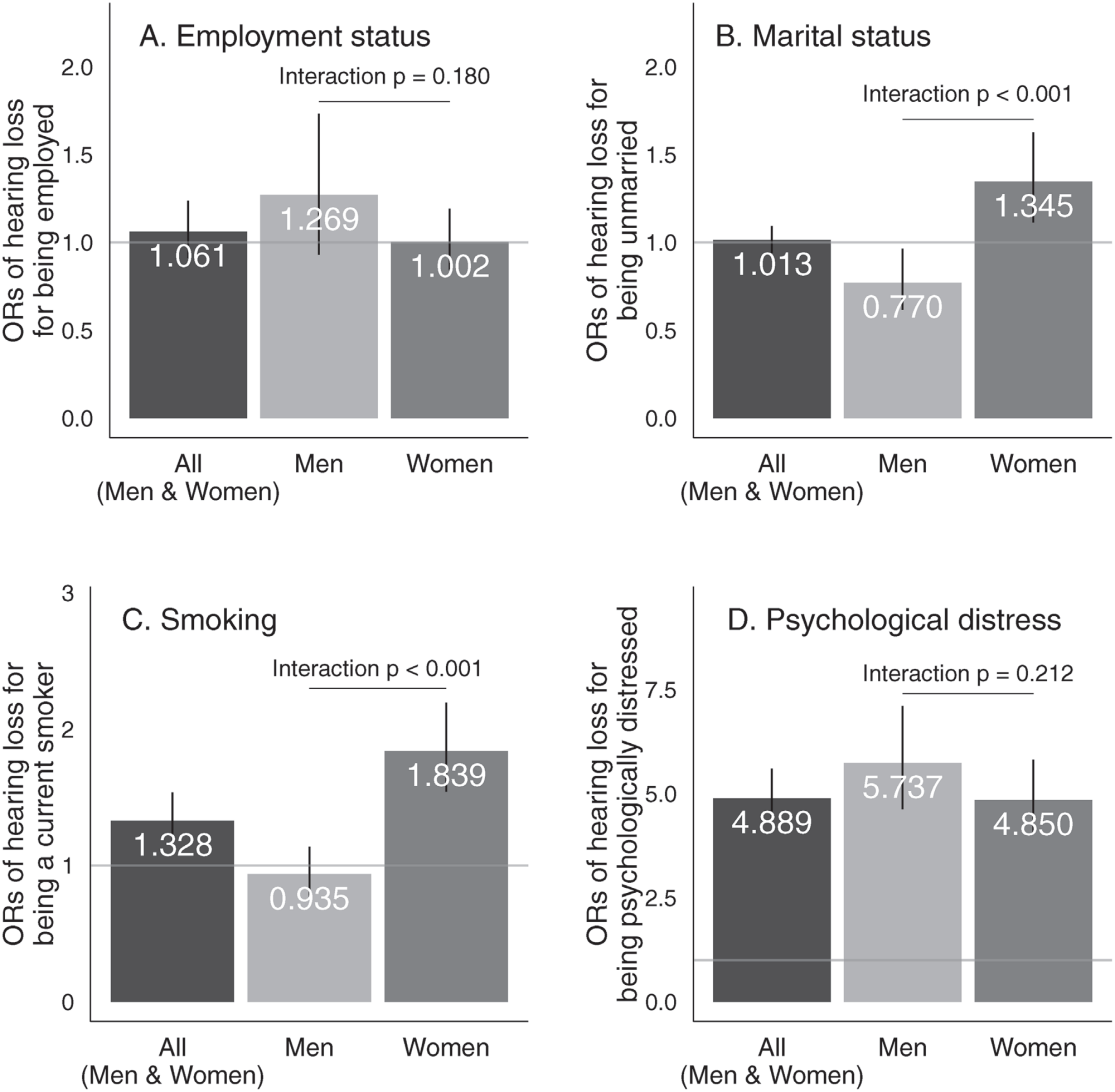
The associations of hearing loss with four outcome variables among both genders, among men, and among women in multivariate analyses. Higher odds ratios (> 1) represent that individual with hearing loss are more likely to be employed (A), unmarried (B), currently smoking (C), and psychologically distressed (D). Bars indicate 95% confidence intervals (95% CIs), and values represent the point estimates for odds ratios (ORs).

Fig. 1 shows the associations between hearing loss and each of the four outcome measures in the multivariate analyses. Fig. 1A shows that hearing loss was not associated with being employed (OR for both genders = 1.061, 95% CI: 0.910–1.237). There was no gender difference in the association (interaction *p* = 0.180).

Fig. 1B shows that hearing loss was not associated with being married (OR for both genders = 1.013, 95% CI: 0.940–1.092). However, after stratification by gender, in men, hearing loss was negatively associated with being unmarried (OR = 0.770, 95% CI: 0.616–0.963), while in women, hearing loss showed a positive association with being unmarried (OR = 1.345, 95% CI: 1.114–1.626, and interaction *p* < 0.001). After excluding the rare cases of widowed status (0.2%) and separated status (3.3%) from the data, the regression results did not change substantially in men or women (OR for both genders = 0.990, 95% CI: 0.913–1.074), among men (OR = 0.734, 95% CI: 0.583–0.924), or among women (OR = 1.246, 95% CI: 1.018–1.525, and interaction *p* < 0.001). This indicates that the results for an unmarried status reflect those for a never married status in our young working-age study population.

Fig. 1C shows that hearing loss was positively associated with current smoking (OR for both genders = 1.328, 95% CI: 1.149–1.536). However, after stratification, this association was found among women (OR = 1.839, 95% CI: 1.540–2.195), but not men (OR = 0.935, 95% CI: 0.770–1.137, and interaction *p* < 0.001). These results reflect that men had a high prevalence of being current smokers regardless of hearing condition (51.6% vs. 50.0%), while women with hearing loss had a higher prevalence of being current smokers compared with women without hearing loss (30.3% vs. 19.0%).

Fig. 1D shows that hearing loss was associated with psychological distress (OR for both genders = 4.889, 95% CI: 4.267–5.601). After stratification by gender, the association was significant in men (OR = 5.737, 95% CI: 4.632–7.106) and women (OR for = 4.850, 95% CI: 4.043–5.818, and interaction *p* = 0.212).

## Discussion

To our knowledge, this is the first reported study providing evidence of the social and health conditions among people living with hearing loss in Japan. We found that hearing loss was associated with several social and health measures. First, there was no association between hearing loss and employment status. Second, women with hearing loss were more likely to be unmarried, while men with hearing loss were more likely to be married. Third, women with hearing loss were more likely to be current smokers, but this was not observed in men. Finally, those with hearing loss were more likely to have psychological distress in both men and women. The most importantly, the significant effect modification by gender on the hearing-loss-marriage association and the hearing-loss-smoking association has never reported previously; our study first reports this potential pitfall in public and health policies, as far as we know.

Regarding employment status, we saw no significant association. Our findings thus differ from those of previous studies in the U.S. [44,45] in which individuals with hearing loss were more likely to be unemployed than those with no hearing loss. These results may reflect the Handicapped Person’s Employment Promotion Act in Japan (since 1960), which requires companies to hire a certain percentage of persons with disabilities. A previous study from Japan indicated that individuals with hearing loss were more likely to leave and change their jobs [46], and thus we need to interpret these results carefully given the simple measure of employment status used (yes/no), which may mistakenly overstate the effects of the Handicapped Person’s Employment Promotion Act. Indeed, an additional analysis indicates that having hearing loss is positively associated with being employed among unmarried women (OR = 1.266, 95% CI = 0.957–1.674), while having hearing loss is not associated with being employed among married women (OR = 1.025, 95% CI = 0.805–1.305), which interaction was not significant (*p* = 0.285). This simply implies a possibility that women with hearing loss who have not married yet are motivated or forced to stay in employment, which we also need to interpret carefully.

Regarding marital status, the results revealed that young working-age men with hearing loss were more likely to be married, while young working-age women with hearing loss were more likely to have never been married. Although a previous study from the U.S. indicated that individuals with hearing loss were more likely to be unmarried [44], gender differences were not assessed. Thus, there is a lack of literature suggesting potential explanations for the gender differences that we found in the present study.

Here, we propose three potential explanations in the Japanese context. First, the gender difference in the prevalence of early-onset hearing loss and assortative mating patterns in those with hearing loss may explain the results. Our data showed a higher prevalence of hearing loss among women. Moreover, because those with hearing loss were reported to more frequently marry partners with hearing loss [47], the excess number of women with hearing loss may make it difficult for them to find a partner in Japan.

Second, men with hearing loss may find a marriage partner more easily than women with hearing loss, given the current situation surrounding people living with disabilities in Japan. A larger number of women (without hearing loss) are engaged in social welfare activities, such as supporting people living with disabilities. For example, 80% of employees in welfare industries in Japan are women [48], and 90% of sign language interpreters in Japan are women [49,50]. Thus, men with hearing loss arguably may have a better opportunity to meet a woman who understands the situation of those living with hearing loss. This may also explain why men with hearing loss were more likely to be married in the present study.

Third, the stigma towards women with hearing loss may still persist in relation to the ideology of eugenics in Japan [51]. The Japanese government approved the legalization of abortion and the Eugenic Protection Law in 1948 (until 1996), which permitted induced abortion and sterilization for health reasons, *including hearing loss* [52]. It had been rumored that Japanese women with hearing loss were discouraged from getting married or forced to not get pregnant (or to get an abortion). There is little literature describing the historical perspectives on how women with hearing loss suffered under this legislation, and thus caution and more information are needed regarding this explanation.

Regarding smoking behavior, the excess risk by hearing loss was found among women (30.3% [with hearing loss] v.s. 19.0% [without hearing loss]), but not among men (50.0% [with hearing loss] v.s. 51.6% [without hearing loss]) in 2007 (please refer to Table 1; and the regression analysis supported this argument). In contrast, a previous study using a roughly comparable population in 1997 shows that the excess risk by hearing loss on smoking behavior was not found among women (12.2% [with hearing loss] v.s. 14.5% [without hearing loss]) or among men (30.6% [with hearing loss] v.s. 56.1% [without hearing loss]) [53]. Since this previous study used a small sample size (N = 126 college students with hearing loss living in a college dormitory) [53], the comparison between the two studies requires a careful operation. Nevertheless, a potential rise of the smoking prevalence among individuals with hearing loss during the decade may be an unfound public health concern in Japan. This can relate to the psychosocial pathways among them (please refer to the next paragraph), and relate to the other possibility that smoking cessation and prevention programs are difficult to reach those with hearing loss (e.g. acquiring appropriate information from the campaigns requires a hearing skill) [7].

Psychological distress was found to be associated with hearing loss, consistent with previous studies [8–10,54]. Previous studies proposed several potential mechanisms to explain the progression from hearing loss to mental illness [8,10]: 1) access to effective communication, 2) socioeconomic environment in relation to stigma and discrimination, and 3) less access to mental health services. First, individuals with hearing loss often do not have access to medical care with effective communication (e.g. reaching healthcare professionals having knowledge of hearing loss, or certified interpreters with health literacy) [55,56]. Second, it has been reported that working adults with hearing loss frequently face negative emotional situations (e.g., stigma and discrimination), leading to a negative physical and mental health status, including social isolation, depression, irritability, and feelings of inferiority [57,58]. Third, individuals with hearing loss often do not have sufficient knowledge about mental illnesses, so they may come to distrust the mental health system, resulting in less frequent access to mental healthcare [59]. Moreover and more generally, individuals with hearing loss can face various kinds of issues and hassles in their daily life [4,8]. Although the gender interaction was not statistically detected in psychological distress, men with hearing loss may have an additional source of psychological distress (e.g. a difficulty in becoming a successful bread-winner, where the Japanese society still wants men to be).

Although we used a large sample from a nationally representative survey, the present study has several limitations. First, our measurement of hearing loss was self-reported, and thus, potential bias in the measurements could generate false-positive associations between hearing loss and the outcome variables. Although the implications from previous reports are not consistent, they grossly suggest that self-reported hearing loss may tend to underestimate the state of hearing loss [60,61]. Second, the 2007 LCPHW is a cross-sectional study, and therefore we need to be careful of any causal interpretation of the reported associations. However, the argument induced by the “reverse causality” is not persuasive clinically: being unmarried, unemployed, or a smoker causes hearing loss. In the 2007 LCPHW, the prevalence of hearing loss from 20 to 39 years of age was almost at a consistent level (0.7–0.8%), and early-onset hearing loss is unlikely to be cured later, suggesting that hearing loss occurred before the four social and health measures. Indeed, lifestyle stress related to these social conditions may induce hearing loss (e.g. psychogenic hearing loss), but this would not likely explain the reported gender differences. Third, several other related measures such as self-rated health and the frequency of physician visits were available in the 2007 LCPHW. However, we didn’t analyze these measures because hearing loss could be an aspect of self-rated health, and hearing loss might require scheduled hospital visits for follow-up at an otolaryngology department. Moreover, the physical disability certificate under services and supports by the Services and Supports for Persons with Disabilities Act allows individuals with severe/profound hearing loss to gain easier access to medical care (no out-of-pocket medical expenditures). Therefore, the impact of early-onset hearing loss on other health conditions and health care use can be the direction of the future research, in which the data to distinguish hearing-loss-related health loss or physician visits from non-hearing-loss-related health loss or physician visits. Fourth, potential confounding factors, which were not measured in the 2007 LCPHW, may at least partially explain the association of early-onset hearing loss for each of the four outcome variables. Such factors include poor maternal diet, maternal alcohol use during pregnancy, premature birth, and noise exposure in childhood, all of which were reported to be associated with early-onset hearing loss in prior literature [62–64]. Fifth and lastly, since status of the physically disabled person’s certificate was not available in the 2007 LCPHW, we could not directly distinguish individuals with severer hearing loss from those with mild or moderate hearing loss, or examine the effect of the formal program. Further more precise original survey will be needed to cover these limitations.

In summary, this study revealed that social and health burdens among individuals living with hearing loss appeared particularly in women. Young working-age women with early-onset hearing loss in Japan are more likely to be unmarried (or never married), smokers, and psychologically distressed than women without early-onset hearing loss. This may be because they have a larger number of unmet needs in their daily lives, and because those with mild to moderate hearing loss have been overlooked in public and health policies and even in public health research. Although the implications of this study cannot be easily stated, the general aims of the CRPD may not be achieved only by legislation.

Future initiatives can include recruitment of professional female counseling staffs in the local government offices, training of women with hearing loss themselves as counselors (peer support), and construction of strong networks of young working-age women with/without hearing loss to get together and share their experiences (social safety net). This study illustrates that many aspects of people with disabilities—job, marriage, smoking, and mental health—require special attention and multidimensional approach by policy makers and all involved. Such attention and approach will accelerate the social change of Japan, and also contribute to realizing the aims of CRPD.
